# Relative Role of Flower Color and Scent on Pollinator Attraction: Experimental Tests using F1 and F2 Hybrids of Daylily and Nightlily

**DOI:** 10.1371/journal.pone.0039010

**Published:** 2012-06-15

**Authors:** Shun K. Hirota, Kozue Nitta, Yuni Kim, Aya Kato, Nobumitsu Kawakubo, Akiko A. Yasumoto, Tetsukazu Yahara

**Affiliations:** 1 Department of Biology, Faculty of Sciences, Kyushu University, Fukuoka, Japan; 2 Department of Environmental Science, Faculty of Applied Biological Sciences, Gifu University, Gifu, Japan; The Australian National University, Australia

## Abstract

The daylily (*Hemerocallis fulva*) and nightlily (*H. citrina*) are typical examples of a butterfly-pollination system and a hawkmoth-pollination system, respectively. *H. fulva* has diurnal, reddish or orange-colored flowers and is mainly pollinated by diurnal swallowtail butterflies. *H. citrina* has nocturnal, yellowish flowers with a sweet fragrance and is pollinated by nocturnal hawkmoths. We evaluated the relative roles of flower color and scent on the evolutionary shift from a diurnally flowering ancestor to *H. citrina*. We conducted a series of experiments that mimic situations in which mutants differing in either flower color, floral scent or both appeared in a diurnally flowering population. An experimental array of 6×6 potted plants, mixed with 24 plants of *H. fulva* and 12 plants of either F1 or F2 hybrids, were placed in the field, and visitations of swallowtail butterflies and nocturnal hawkmoths were recorded with camcorders. Swallowtail butterflies preferentially visited reddish or orange-colored flowers and hawkmoths preferentially visited yellowish flowers. Neither swallowtail butterflies nor nocturnal hawkmoths showed significant preferences for overall scent emission. Our results suggest that mutations in flower color would be more relevant to the adaptive shift from a diurnally flowering ancestor to *H. citrina* than that in floral scent.

## Introduction

Hawkmoth pollination has been of continuous interest in evolutionary biology since the time of Darwin [1] because flowers pollinated by hawkmoths have a unique set of floral traits such as pale-colored petals, a sweet floral scent and narrow flower tubes or spurs [2]–[10]. Those flowers exhibiting the set of traits characteristic of hawkmoth-pollination have been referred to as having hawkmoth-pollination syndrome [10], [11] or system [12]–[14]. In contrast, flowers of related species which are pollinated by hummingbirds (*Aquilegia formosa*: [6], e.g., *Ipomopsis aggregata*: [7]), swallowtail butterflies (e.g., *Hemerocallis fulva*: [4]), bees (e.g., *Petunia integrifolia*: [15]) or long tongued flies (e.g., *Disa scullyi*: [16]), have another set of markedly different floral traits including weak scent and strong flower colors such as red and purple. The hawkmoth-pollination system is considered to be derived from diurnal pollination systems [13], [14], [17]. However, it is unclear how the evolutionary shift occurred. To deepen our understanding of this evolutionary shift, we need to know how hawkmoths and other pollinators respond to the changes in visual and olfactory traits of flowers that markedly differ between hawkmoth-pollinated flowers and other types.

Both pale-colored petals and night-scent profiles are characteristic of hawkmoth-pollinated flowers. However, we do not know which trait is the more important for attracting hawkmoths. Several studies have shown how olfactory and visual stimulations play (or do not play) a role in attracting hawkmoths. Balkenius et al. [18] demonstrated that the nocturnal hawkmoth, *Deilephila elpenor*, is more attracted to flower odor than visual display. Raguso and Willis [19] demonstrated that olfactory or visual cues alone can attract a crepuscular-nocturnal hawkmoth, *Manduca sexta,* within 5 m of a flower, although only in combination with a visual display and scent would the moths begin feeding. Klahre et al. [20] then demonstrated that *M. sexta* displays no preference when exposed to conflicting cues of color versus scent. This indicates that color and scent are equivalent cues. Goyret et al. [21], however, demonstrated that *M. sexta* favored the visual target over the odor source when visual and olfactory floral cues were decoupled spatially. They showed a sensory bias for the visual display over the odor plume, suggesting the former to be the ultimate indicator of a nectar source. Furthermore, *M. sexta* can extend their proboscis to scentless feeders [22]. Diurnal Lepidoptera were also studied with respect to visual and olfactory cues. The Indian red admiral *Vanessa indica* depends primarily on color and secondarily on scent during flower visitations [23]. The diurnal hawkmoth, *Macroglossum stellatarum*, strongly preferred the visual source to the odor source [18] and learned the flower odor when the flower colors were not preferred innately [24].

To test the independent effects of visual and olfactory cues upon pollinator behavior, we need to design an experiment in which visual and olfactory traits are not correlated among plants being studied. However, in many flowers including hawkmoth-pollinated ones, color and scent are correlated, making it difficult to isolate their independent effects. To overcome this difficulty, we produced hybrids between a hawkmoth-pollinated species, the nightlily (*H. citrina* var. *vespertina* (H. Hara) M. Hotta) and a butterfly-pollinated species, the daylily (*Hemerocallis fulva* L. var. *aurantiaca* (Baker) M. Hotta).

**Figure 1:**
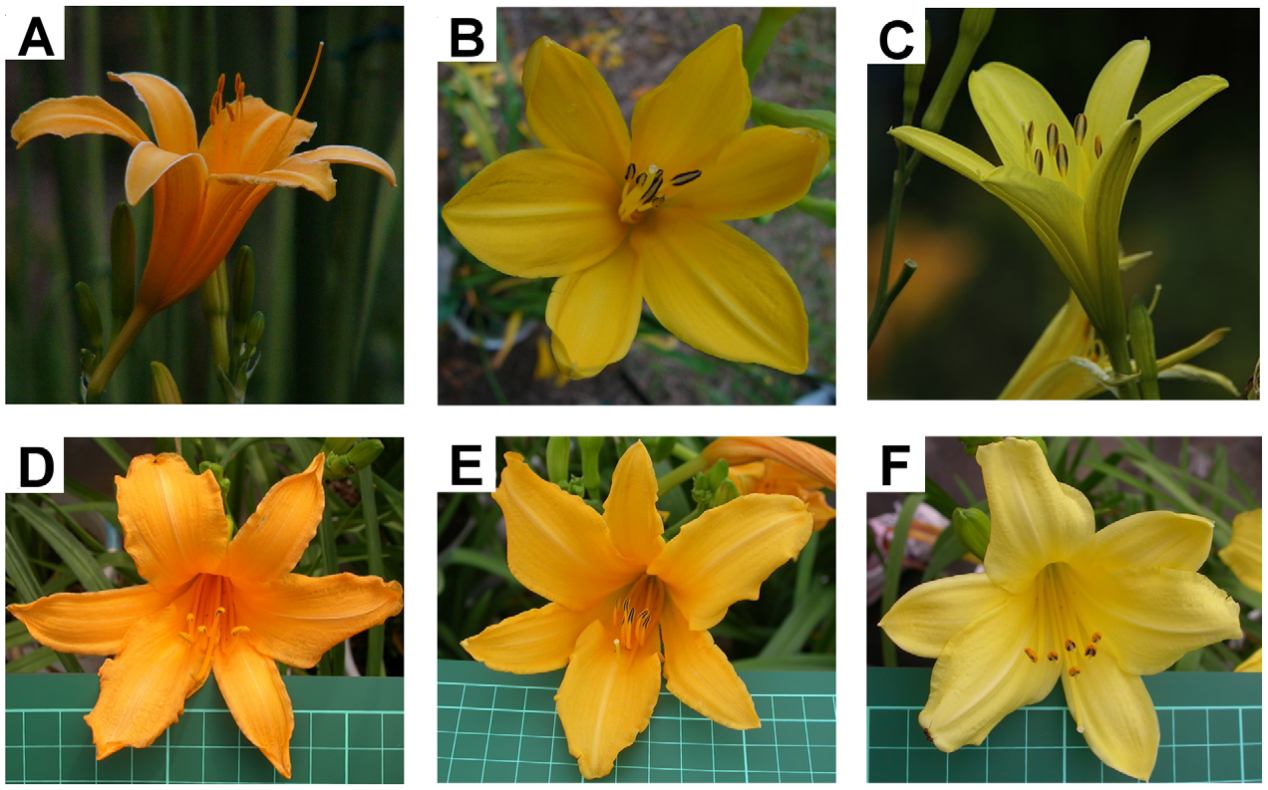
Flowers of *H. fulva* (A), F1 hybrid (B), *H. citrina* (C) and F2 hybrids (D-F).

*H. fulva*, a butterfly-pollinated species, has diurnal, reddish or orange-colored flowers without scent and *H. citrina*, a hawkmoth-pollinated species has nocturnal, yellowish flowers with a sweet scent [4], [25]. According to a molecular phylogenetic study of *Hemerocallis* (Yasumoto et al., unpublished), *H. citrina* is closely related to diurnally flowering species (*H. fulva* and its relative) indicating that nocturnal flowering evolved from diurnal flowering in *Hemerocallis*. Hybrids of two species are highly fertile [26], and floral traits, including those of color and scent, are segregated in F2 hybrids (Fig. 1).

In this study, we conducted a series of field experiments using *H. fulva* and F1 or F2 hybrids to answer the following question: which floral trait, flower color and floral scent, do butterflies and hawkmoths use as dominant cues in the approach of flowers?

## Results

### Petal Color and Floral Scent

**Figure 2:**
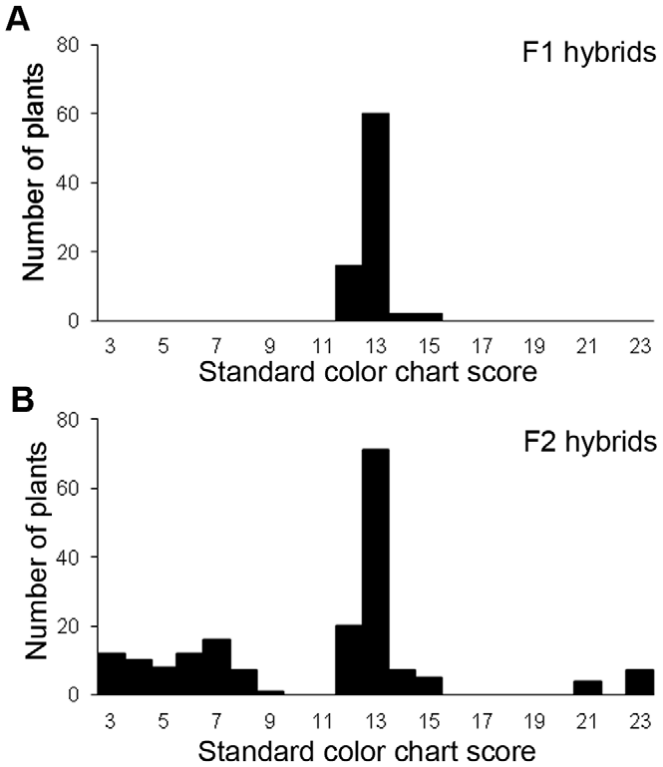
Variation of flower colors in F1 (A) and F2 hybrids (B). The horizontal axis is the standard color chart score. Larger scores indicate reddish color and smaller scores indicate yellowish color. Color chart scores from 2–13 were classified as the yellow group, and color chart scores from 14–23 were classified as the yellow-orange group. The vertical axis is the number of plants.

**Figure 3:**
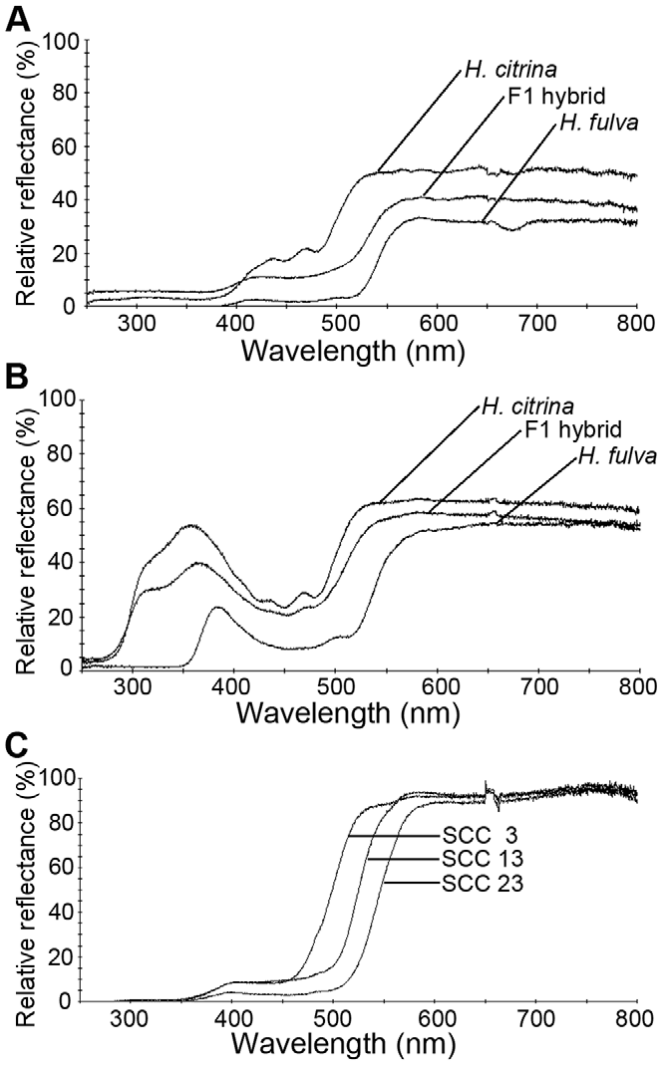
Reflectance spectra of tepals of two *Hemerocallis* species, F1 hybrid and Standard Color Charts. (A) Reflectance spectra of the central part of tepals (upper, *H. citrina*; center F1 hybrid; lower, *H. fulva*). (B) Reflectance spectra of the peripheral part of tepals (upper, *H. citrina*; center F1; lower, *H. fulva*). (C) Reflectance spectra of three representative Standard Color Charts (upper, SCC=3; center SCC=13; lower, SCC=23).

**Figure 4:**
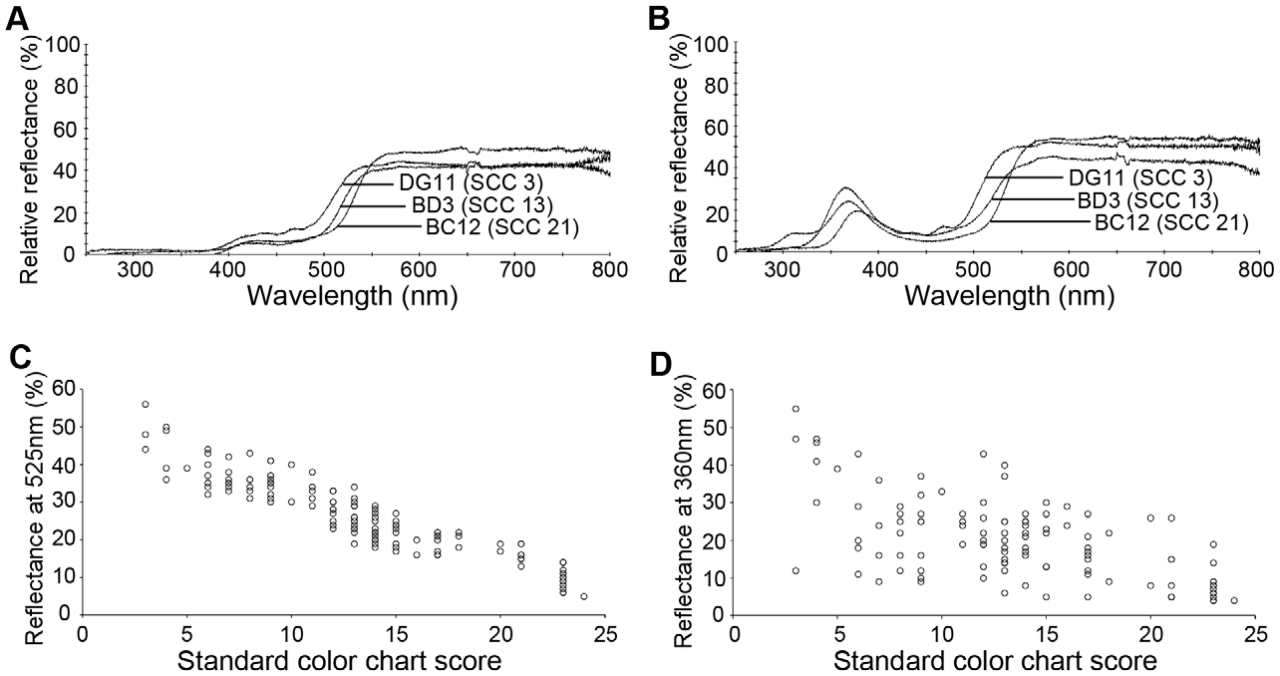
Typical reflectance spectra of F2 hybrids (above) and the relationship score, reflectance (below). (A) Reflectance spectra of the central part of tepals. Three representative F2 hybrids, DG11 (SCC=3), BD3 (SCC=13) and BC12 (SCC=21), are showed. (B) Reflectance spectra of the peripheral part of tepals. (C) The relationship between color chart score and relative reflectance at 525 nm of the central part of tepals. (D) The relationship between color chart score and relative reflectance at 360 nm of the peripheral part of tepals.

The flower color of *H. fulva* was reddish-orange and qualified with the standard color chart (SCC) as SCC 21-23 (Fig. 1A). The flower of F1 hybrids was yellow, qualified between SCC 11-15, and was therefore more yellowish than *H. fulva* (Fig.1B, Fig. 2A), but more orange-colored than *H. citrina* (SCC 3–4, Fig. 1C). The reflectance spectra shown in Fig. 3 (3A, central part of tepals; 3B, peripheral part; upper, *H. citrina*; center F1 hybrid; lower, *H. fulva*) differed notably between the two species at 525 nm corresponding to the peak sensitivity of the green receptor of butterflies and moths (*Papilio xuthus*: 520 nm, [27], *Deilephila elpenor*: 520–525 nm, [28], [29], *Manduca sexta*: 520 nm, [30]). The reflectance spectra of the color chart SCC 3, 13 and 23 largely differed at 525 nm. The reflectance spectra of the peripheral part of tepals largely differed also at 360 nm, corresponding to the peak sensitivity of the UV receptor of butterflies and moths (*P. xuthus*: 360 nm, [27], *D. elpenor*: 345–350 nm, [28], [29], *M. sexta*: 357 nm, [30]). This difference is not significant in the central part because tepals of *Hemerocallis* have a nectar guide in the central part that absorbs ultraviolet light. F2 hybrids showed high variability in flower color (SCC 3–23; Fig. 1D,E, F, Fig. 2B). Fig. 4A, B shows three typical reflectance spectra of F2 hybrids, DG11, BD3, BC12, qualified as SCC 3, 13, and 21, respectively. The reflectance at 525 nm (y) was correlated with SCC scores (x) as y=–1.73x+49.43 (*P*<0.001; Fig 4C), and the reflectance at 360 nm was also correlated with SCC scores as y=–1.10x+35.20 (*P*<0.001, Fig. 4D). However, the latter correlation was more dispersed than the former, and SCC scores mostly reflect the difference of the reflectance at 525 nm.

**Figure 5:**
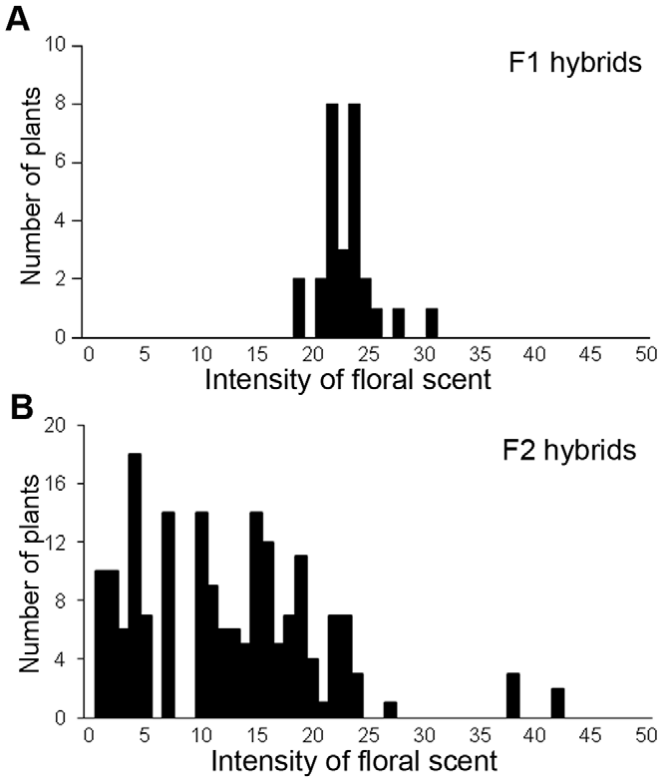
Variation of fragrance intensity in F1 (A) and F2 hybrids (B). The horizontal axis is the intensity of floral scent measured with a handheld odor meter. The odor meter can show relative intensity of scent in an arbitrary scale. All data sets were measured by the same odor meter for reproducibility. The vertical axis is the number of plants.

**Figure 6:**
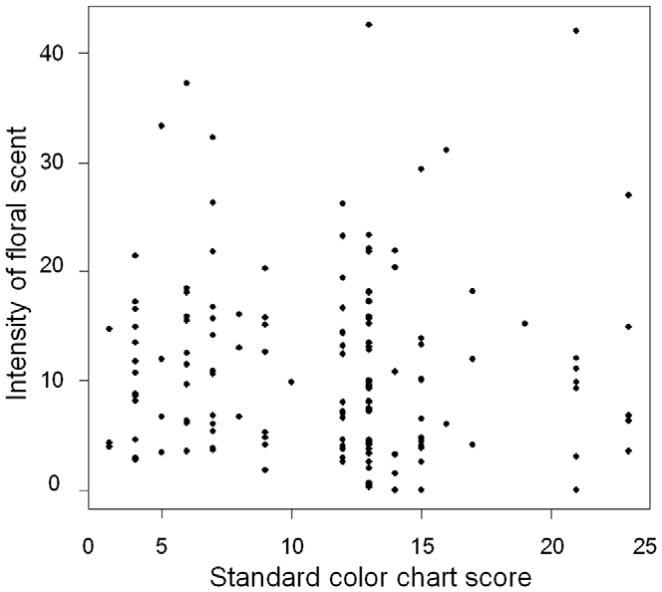
Relationship between flower color and fragrance in F2 hybrids. The horizontal axis is the standard color chart score. The vertical axis is the intensity of floral scent measured with the odor meter. A Pearson's product-moment correlation coefficient is –0.0336 (*P*=0.684).

Flowers of *H. fulva* had little or no recognizable floral scent (intensity of scent varied from 1.1 to 3.8, with a mean ± SE, 2.30±0.28, n=49). F1 flowers had a sweet scent (19.4 to 31.7, 22.5±1.31, n=9, Fig. 5A), which was not significantly weaker than that of *H. citrina* (18.0 to 47.6, 26.8±3.31, n=9; *t* test, *t*=1.22, df=16, *P*=0.23). F2 flowers had large variation in floral scent (0.3 to 42, 12.2±1.13, n=54; Fig. 5B). In F2 hybrids, there was no significant correlation between flower color and intensity of scent (Pearson's product-moment correlation coefficient =–0.0336, *P*=0.684, Fig. 6).

### Types of Pollinator

We arranged three types of experimental arrays in the experimental field and observed pollinator foraging. In experiment 1, the experimental array was consisted of 24 and 12 pots of *H. fulva* and F1 hybrids, respectively. In experiment 2, the experimental array was consisted of 24 and 12 pots of *H. fulva* and F2 hybrids, respectively. By mixing 12 plants of F1 or F2 hybrids with 24 plants of *H. fulva*, our experiments mimicked the situations in which mutants for either or both flower color and floral scent appeared in a lower frequency within an ancestral population like *H. fulva*. In addition to this mixed array, we used an array consisting only of *H. fulva* in order to observe the foraging behavior of hawkmoths in ancestral population.

**Figure 7:**
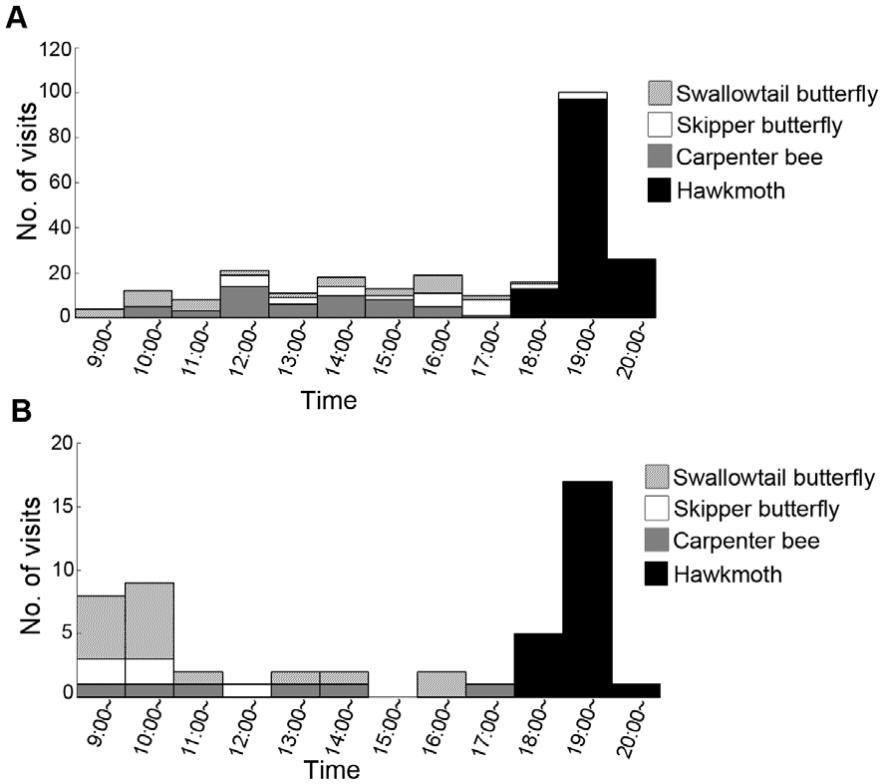
The number of four groups of flower visitors observed in each time zone. The number of visitors were pooled during the observation period of experiment 1 (A) and experiment 2 (B).

We observed a total of 930 pollinator visits in experiment 1, and 108 pollinator visits in experiment 2. The flower visitors were swallowtail butterflies (*Papilio xuthus*, *P. memnon thunbergii* and *P. helenus nicconicolens*), nymphalid butterflies (*Argyreus hyperbius hyperbius*), skipper butterflies (*Parnara gutttata*), hawkmoths (*Theretra oldenlandiae* and *T. silhetensis*), and carpenter bees (*Xylocopa appendiculata circumvolans*). Visits of swallowtail butterflies and hawkmoths amounted to 272 (26.2%) and 520 (50.1%) of the total of 1038 visits, respectively. Hawkmoths were observed exclusively in the evening, while the other species, butterflies and bees, were observed in the daytime (Fig. 7). Swallowtail butterflies were observed from 9:00 to 19:00. In experiment 1, they observed every period of daytime. In experiment 2, they were observed frequently during the morning. During the 15 days of experiment 1, swallowtail butterflies were observed on 10 days, hawkmoths on 12 days (Table 1), and both on 9 days. During these 9 days, 6.63±2.43 flowers of *H. fulva* were visited per day only by butterflies, 6.25±2.44 only by hawkmoths and 7.13±1.88 by both butterflies and hawkmoths. For flowers of F1 hybrids, 0.75±0.25 were visited only by butterflies, 6.88±0.52 only by hawkmoths and 2.50±0.71 by both butterflies and hawkmoths. During experiment 2, swallowtail butterflies were observed in 8 days, hawkmoths in 8 days, and both in 5 days. During these 5 days, the number of *H. fulva* flowers visited per day by either or both of butterflies and hawkmoths was 3.80±1.53 (only butterflies, mean ± SE), 1.20±0.73 (only hawkmoth) and 0.20±0.20 (both butterflies and hawkmoths). The number of F2 hybrids’ flowers visited per day by either or both of butterflies and hawkmoths was 0.40±0.24 (only butterflies), 1.80±0.66 (only hawkmoth) and 0 (both butterflies and hawkmoths). In the *H. fulva* only array, we observed 6 foraging bouts and 101 visits of hawkmoths during 5 days.

**Table 1:** Statistics for four pollinator groups in field experiments.

	Experiment 1 (2006)			Experiment 2 (2007)		
Pollinator type	Pollinators	Visitations	Days	Pollinators	Visitations	Days
Swallowtail butterfly	38	227	10	16	45	8
Nymphalid butterfly	8	16	4	1	2	1
Skipper butterfly	32	35	5	5	6	2
Hawkmoth	136	482	12	23	38	8
Carpenter bee	52	170	8	6	17	5

“Pollinators” indicate that the number of individuals in each pollinator group that visited flowers of *H.fulva* and F1 hybrid (Experiment 1, 2006) and *H. fulva* and F2 hybrids (Experiment 2, 2007) during the 15 days of observations. We regarded any pollinator which foraged in the experimental array and then left as one individual, and counted the number of pollinators that foraged in the array. “Visitations” indicate the total times each pollinator in each pollinator group visited flowers of *H.fulva* and hybrids. “Days” indicate the number of days on which one or more pollinators in each group were observed.

### Pollinator Preference

#### Experiment 1

**Figure 8:**
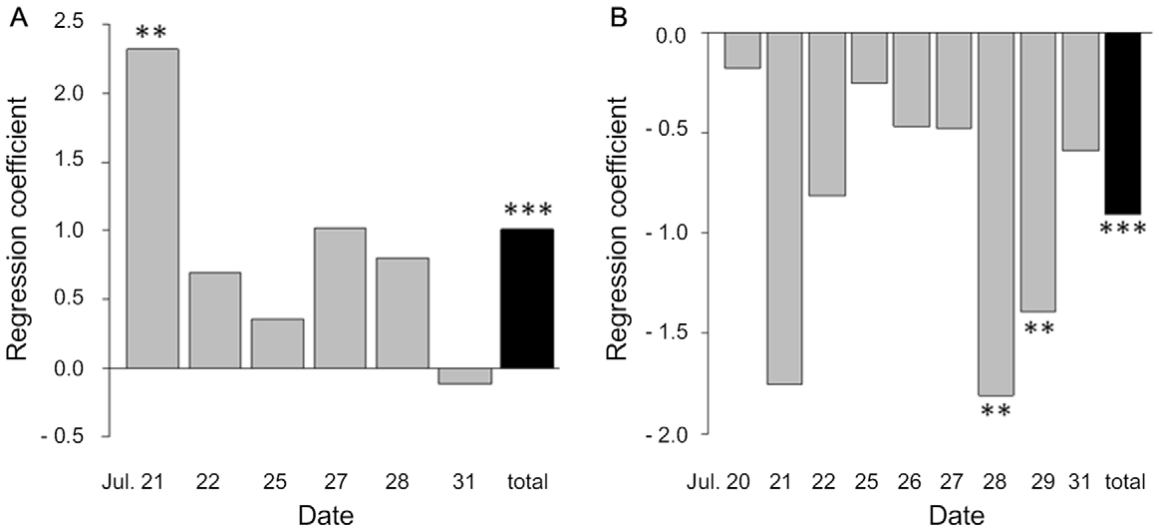
Partial regression coefficients of frequency of pollinator visits on floral types, *H.fulva* or F1 hybrid. Daily results (gray bars) and total results (black bars) are shown along the horizontal axis in the order of date. The positive regression coefficient means that the pollinators prefer *H. fulva*. Conversely the negative regression coefficient means that the pollinators prefer F1 hybrids (***, *P*<0.001; **, *P*<0.01; *, *P*<0.05; after Bonferroni correction, (A) swallowtail butterflies, n=324, (B) hawkmoths, n=360).

GLMM analysis of visitation data over the observation period showed that swallowtail butterflies significantly preferred flowers of *H. fulva* to flowers of F1 hybrids (β±SE=1.09±0.18, df=356, z=5.93, *P*<0.001; Fig. 8A), while hawkmoths had an overall significant tendency to prefer flowers of F1 hybrids to flowers of *H. fulva* (β±SE=–0.91±0.08, df=428, z=–10.81, *P*<0.001; Fig. 8B). When visitation data were tested on each day, trends were significant only on 3 days: 21 July for swallowtail butterflies and 28 and 29 July for hawkmoths due to the smaller sample size (gray bars of Fig. 8).

Next, we examined whether pollinators showed the significant constancy of floral choice. The frequency of visits to *H. fulva* (Hf) and the frequency of visits to F1 were designed as p and q respectively below. The expected frequencies of Hf–Hf, Hf–F1, F1–Hf and F1–F1 based on the single-plant-visit frequency were p^2^, pq, pq and q^2^, respectively. For swallowtail butterflies, the expectation generated from the single-plant-visit frequencies was not rejected, indicating no evidence of floral constancy (Table 2). For hawkmoths, the expectation was rejected; they made significantly more homotypic plant-to-plant (both Hf–Hf and F1–F1) movements than expected.

**Table 2:** Plant-to-plant transitions made by pollinators in plots containing *H. fulva* and F1 hybrids.

	Plant-to-plant transition matrices							
Pollinators	Expected			Observed			G (df=1)	*P*
Butterflies		*H. fulva*	F1		*H. fulva*	F1		
	*H. fulva*	143	24	*H. fulva*	147	23		
	F1	24	4	F1	21	4		
					sum=195		0.538	0.463
Hawkmoths		*H. fulva*	F1		*H. fulva*	F1		
	*H. fulva*	94	107	*H. fulva*	143	72		
	F1	107	121	F1	77	137		
					sum=429		46.034	*P*<0.001

The hypothesis tested here is that plant-to-plant movements are a simple extension of single-flower preference. Expected plant-to-plant movement frequencies are based on single-flower-visit preference and are round to whole numbers for presentation. If homotypic movements were more frequent than expected, then that provides evidence for floral constancy. The direction of movements is from the species listed on the left of each matrix to the species listed above.

#### Experiment 2

The all interaction terms between flower color and scent in the GLMM analyses were not significant (swallowtail butterfly over the observation period: β±SE=–0.18±0.28, df=279, z=–0.65, *P*=0.518; hawkmoth over the observation period: β±SE=0.31±0.23, df=280, z=1.35, *P*=0.177). Thus, in this study, the final models did not include the interaction terms, and the effects of flower color were independent of the effects of scent intensity.

**Figure 9:**
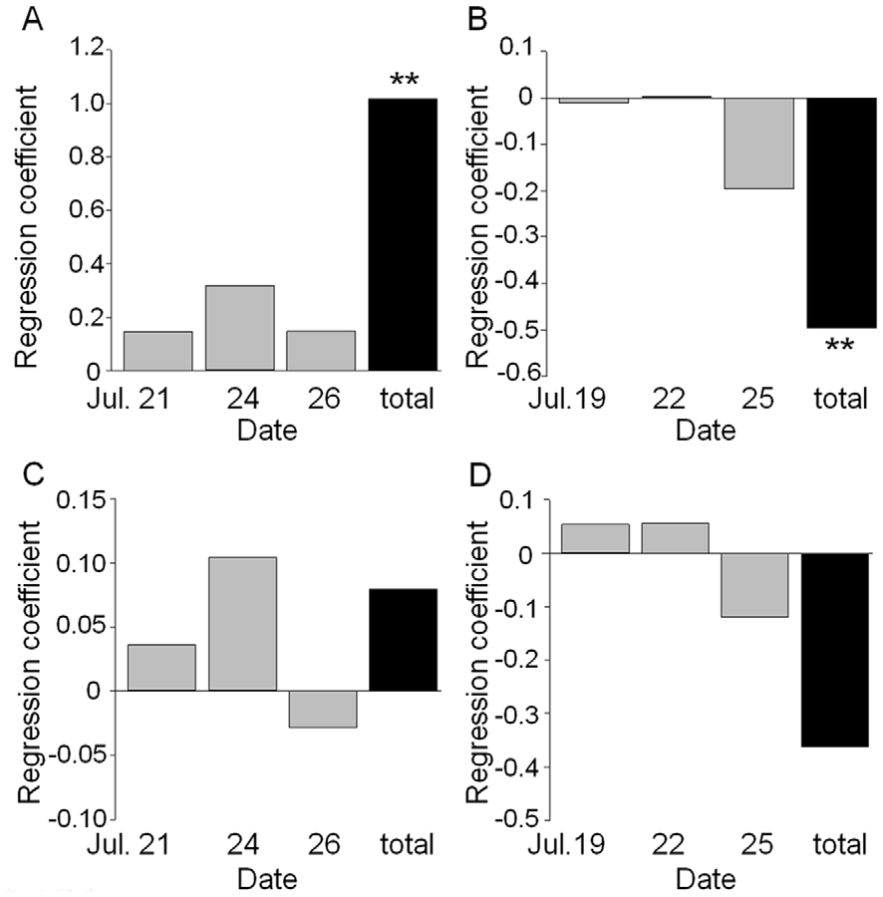
Partial regression coefficients of frequency of pollinator visits on flower color and scent intensity. Gray bars represent the daily results and black bars represent total results (**, *P*<0.01; *, *P*<0.05 after Bonferroni correction. panel A, C: swallowtail butterflies, n=285, panel B, D: hawkmoths, n=286). In flower color (A, B), the positive regression coefficient means that the pollinators prefer reddish flowers to yellowish flowers. Conversely the negative regression coefficient means that the pollinators prefer yellowish flowers to reddish flower. In floral scent (C, D), the positive regression coefficient means that the pollinators prefer the flowers with stronger scent. The negative regression coefficient means that the pollinators prefer the flowers with weaker scent.

Swallowtail butterflies showed significantly higher proportion of visits to reddish flowers (β±SE=1.02±0.34, df=280, z=3.01, *P*=0.003; Fig. 9A), whereas hawkmoths showed significantly higher proportion of visits to yellowish flowers (β±SE=–0.50±0.16, df=281, z=–3.03, *P*=0.002; Fig. 9B). In the analyses of each observation day, swallowtail butterflies also displayed a similar but non-significant tendency to prefer red flowers (Fig. 9A). On the other hand, hawkmoths showed a large variation in color preference within each observation day (Fig. 9B). For floral scent, both butterflies and hawkmoths showed no significant preference (swallowtail butterflies: β±SE=0.08±0.23, z=0.351, *P*=0.726; hawkmoths: β±SE=–0.362±0.215, z=–1.68, *P*=0.092; Fig. 9C, D).

GLM analysis of the illumination effect on hawkmoth preference showed that the preference of hawkmoths was independent of the illumination (β±SE=0.128±0.333, *x^2^*=–0.145, *P*=0.703).

## Discussion

Swallowtail butterflies and hawkmoths showed preference for reddish flowers and yellowish flowers, respectively, but they did not show significant preference to scent intensity (Fig. 9). Therefore, we were able to demonstrate that swallowtail butterflies and hawkmoths primarily use color as a cue for flower visits, with contrasting preferences toward reddish and yellowish color, respectively. Our finding agrees with previous observations that wild butterflies prefer red and orange flowers (e.g. [31]), while hawkmoths prefer white and yellow flowers (e.g. [12]). Our conclusion is unique in that evidence is obtained from F2 hybrids in which color and scent intensity are segregated. On the other hand, our evidence has still some limitations. First, flower visit time of butterflies and hawkmoths largely differed on a day and the sunlight spectra change over time, which might have affected the appearance of flowers for pollinators visiting the flowers at different time of a day. The swallowtail butterfly, *Papilio xuthus,* exhibits color constancy when searching for food [32]. Thus, the effect of sunlight spectra change on swallowtail butterflies is probably very small. The hawkmoth, *Deilephila elpenor*, also exhibits some degree of color constancy though the accuracy of discrimination between yellow and orange depends on illumination [33]. In this study, the preference of hawkmoths on petal color was independent of the halogen-lamp illumination. However, we need to evaluate the preference on color in the darker condition, such as moonlight or starlight, using infrared video cameras. Second, the reflectance spectra at both 360 nm and 525 nm were correlated with SCC scores (Fig. 4C, D). Thus, we cannot exclude a probability that pollinators recognized petal color based on the UV reflection in addition to the reflectance in the green and red region.

More recent studies showed that swallowtail butterflies have an innate color preference toward yellow and red than blue and green [34], [35], while hawkmoths have an innate preference for blue and weaker innate preferences for violet and yellow [36]. On the basis of these points, we discuss the following questions: Why did swallowtail butterflies prefer reddish flowers despite their innate preference to both yellow and red? Likewise, why did hawkmoths prefer yellowish flowers? Recent experimental studies on butterflies [35]–[38] and hawkmoths [24], [39]-[41] showed that these insects can be trained to switch their preference to other colors by learning an association between a certain color and a nectar reward. Thus, it is plausible that swallowtail butterflies and hawkmoths switched their preference to red and yellow colors, respectively, in this manner.

Most insects lack a “red” receptor [42], but swallowtail butterflies have a “red” receptor and can see reddish flowers [43]. Recent studies showed that red light (up to 650 nm) can stimulate a “green” (540 nm) receptor for the majority of bees, provided that the light source is sufficiently intense [44]. However, many bees, whilst retaining the ability to recognize red flowers despite their lack of possession of a “red” receptor, require significantly longer to find these red flowers as compared to those of other colors [45]. Thus, it is more costly for bees to feed on red flowers than on other flowers with more conspicuous colors. This is probably the reason why many bees tend not to feed on red flowers [46]. In contrast, for butterflies, it is probably a better strategy to forage on reddish flowers which are seldom visited by bees.

Naïve hawkmoths probably visited yellowish flowers more than reddish flowers, because hawkmoths have an innate preference for blue and weaker innate preferences for violet and yellow [36]. Furthermore, innate preference is often kept even after learning of another color [47]. However, in this study, the majority of observed hawkmoths were probably experienced rather than naïve. In this situation, the availability of nectar rewards probably affected hawkmoths’ preference. In our experiment, both reddish and yellowish flowers opened in the morning and thus provided nectar rewards similarly. However, swallowtail butterflies, and possibly diurnal bees, might have depleted nectar from the flowers of *H. fulva* during daytime, due to their strong preference for reddish flowers. Therefore, yellowish flowers would have provided larger, less depleted, amounts of nectar than reddish flowers in the evening. For hawkmoths, therefore, it would be a better strategy not to forage on depleted reddish flowers but upon undepleted yellowish flowers. In fact, hawkmoths showed significantly higher proportion of visits to yellowish flowers in experiment 2. In addition, further experiments using fresh (non-depleted) flowers of *H. fulva* showed that hawkmoths shifted to visit those flowers (Hirota et al. in preparation).

Hawkmoths showed significant floral constancy by visiting flowers of similar color more frequently within a foraging bout in experiment 1. It is notable that not only F1–F1 (between yellowish flowers) movements but also Hf–Hf (between reddish flowers) movements were frequently found (Table 2). This observation could be explained by individual hawkmoth’s shifts of the preference or by variability of preference among hawkmoth individuals. Neither possibility was rejected because we did not discriminate individual hawkmoths. However, hawkmoths can learn association of flower color with the presence of nectar rewards [41], suggesting that they are more likely to select the color of flowers having more nectar rewards within a single foraging bout.

It was unexpected that hawkmoths did not show any significant preferences regarding scent intensity (Fig. 9D). Furthermore, hawkmoths visited and foraged in the array consisted of *H. fulva* only. Three hypotheses, not mutually exclusive, can explain this finding. First, hawkmoths may have a bias of visual stimuli over and against olfactory stimuli [21]. Second, effects of floral scent might change depending on the distance to a pollinator. To be more precise, strong floral scent might attract pollinators from significant distances to approach the general area, but have no or a weak subsequent effect on pollinators, beyond their arrival in the vicinity. However, according to a study on hawkmoths [19], both isolated visual cues and isolated odor cues alone proved attractive within 5 m of a flower. In this study, at the presence of floral scent hawkmoths were seen to initiate feeding even at the most visually conspicuous flowers. On the other hand, our experimental method allowed us to detect the effect upon pollinators that arrived at the patch, but not the effect toward pollinators that were further distances away from the patch. Third, scent composition, rather than scent intensity, plays a more definitive role in determining hawkmoth preference. F2 flowers may have a scent composition different from *H. citrina* as a result of hybridization and segregation; and it could be that it is that composition which may be less effective to attract hawkmoths. As already noted, the odor meter may have been insufficient to measure a scent stimulus toward hawkmoths appropriately, due to its lack of sensitivity to changes in the composition of scents. Hawkmoths can learn association of floral scent with the presence of nectar rewards [48], [49]. We need to examine the effects of the scent composition on hawkmoth attraction.

Irrespective of the reason why hawkmoths did not show significant preference for scent intensity, our results suggest that mutations of flower color, rather than, flower scent, could trigger a shift of a pollination system in the genus *Hemerocallis*. This conclusion corresponds to that of previous studies on *Mimulus* [50] and *Petunia* [8], which demonstrated the pollinator shift under a single-gene change of flower color. Our experiments reconstructed, although not exactly, an initial stage of the divergence from diurnally flowering ancestors towards the situation of *H. citrina*. As a result, we showed that plants (“mutants”) having yellowish flowers attracted more hawkmoths in the evening while swallowtail butterflies preferred reddish flowers during daytime. Thus disruptive selection of flower color would be expected under the cases of assortative mating that would occur in partly isolated populations where either swallowtail butterflies or hawkmoths are more abundant. The experimental population with F2 hybrids we developed in this study will provide opportunities to quantify this disruptive selection in further detail.

## Materials and Methods

### Provenance of Experimental Plants

Plants of the butterfly pollinated *H.fulva*, were collected in Haifuku, Hirado island, Nagasaki Prefecture, Japan. *H. citrina* was collected in Tsutsumi, approximately 10 km NE of Haifuku (for details, see [51]). To produce F1 hybrids, *H. fulva* plants were hand-pollinated by pollen of *H. citrina* in 2001 [26]. To produce F2 hybrids, F1 plants were hand-pollinated by pollen of full sibling F1 plants in 2003 and 2004 [25]. All plants were grown in pots in the nursery of the Department of Biology, Kyushu University, Fukuoka, Japan.

Tepals of *Hemerocallis* have a nectar guide in the central part which absorbs ultraviolet light. We recorded flower color of the central part of tepals for all the plants used for experiments by simple matching with the standard color chart (SCC) of the Royal Horticultural Society, London, England. In addition, we measured the reflectance spectra of the central and peripheral part of tepals for representative individuals using a spectrometer (USB2000, Ocean Optics, Inc., USA) in order to confirm the correlation of SCC with the reflectance spectra. The SCCs of the plants used in field experiments were measured before observations commenced. We measured the intensity of floral scent using a handheld odor meter (OMX-SR, Shinyei, Japan). This instrument measures the relative intensity of scent as a resultant vector of two outputs by a pair of semiconductor sensors having unique responses of resistance changes to absorbed volatile chemicals. These semiconductor sensors are very sensitive to changes of scent intensity, but less sensitive to changes in the composition of scents. We used this handheld odor meter because of its convenience of use in collecting multiple data sets in the field [52], [53]. From 15 June to 26 August in 2006 and from 19 July to 2 August in 2007, flower scent intensity was measured immediately after flower opening. The measurements were performed for at least three flowers per plant and then averaged.

### Experimental Design

To examine the preference of pollinators to flowers of *H. fulva* and F1/F2 hybrids, a total of 36 potted plants were arranged in a square reticular pattern of 6×6 with a distance of 50 cm between each pot. In experiment 1, conducted in 2006, we used 24 plants of *H. fulva* and 12 of F1 hybrids to examine whether different pollinator types show different preferences between *H. fulva* and F1 hybrids, depending on the combinations of flower color and scent (*H. fulva*: red flower without scent, F1 hybrids: yellow flower with scent). Because experiment 1 does not allow us to partition the effects of flower color versus scent, we conducted experiment 2 in 2007 using 24 plants of *H. fulva* and 12 plants of F2 hybrids. Due to their high phenotypic variance in color and scent (Fig. 2, 5), F2 flowers allowed us to evaluate the relative importance of flower color and scent to visitation frequencies of different pollinator types. By mixing 12 plants of F1 or F2 hybrids with 24 plants of *H. fulva*, our experiments mimicked the situations in which mutants for either or both flower color and floral scent appeared in a lower frequency within an ancestral population like *H. fulva*. In addition to this mixed array, we used an array consisting only of *H. fulva* in order to observe the foraging behavior of hawkmoths toward ancestral population. No *H. citrina* only array was used in order to observe butterfly response for *H. citrina* because the flower opening time of *H. citrina* peaks at 18:30 [25] and the opportunity for butterflies to visit its flowers is therefore much restricted. In all experiments, we randomly selected one flower and cut off all remaining ones if the plant had two or more flowers. We replaced some of the 36 plants with new ones day by day because the longevity of a flower is only half a day, and each individual plant does not flower every day. The experimental array was placed inside the experimental field of the Department of Biology, Kyushu University where swallowtail butterflies and hawkmoths were common. No specific permits were required for the described field studies. Experiments 1 and 2 were observed from17–31 July 2006 and 19 July–2 August 2007, respectively, while the *H. fulva* only array was performed from 4–9 August 2011. These dates correspond closely to the peak flowering times in natural populations of the two *Hemerocallis* species.

We commenced the experiments each day from 09:30 until 20:30. With regard to the *H. fulva* only array (the observation of the foraging behavior of hawkmoths toward ancestral population), we started observations at 18:30 and stopped at 20:30. In *H. fulva*, start-to-close time varied from 18:00 to 20:30 with a peak at 20:30 [25]. F1 hybrids also start to close after sunset. F2 hybrids were divided into two classes for flower opening times, 00:00 to 12:00 and 12:00 to 24:00 hours and had a wide range of start-to-close time. We used the F2 hybrids that flowered in the morning. The plants that started to close flowering before the end of observation were replaced with new ones having a still opening flower. We used a High-Definition Video Camera Recorder XL H1 (Canon, Tokyo, Japan) to record video images of experimental arrays. This video camera delivers high performance and outstanding digital image quality with which we could exactly follow the movements of pollinators from one flower to another before sunset. At thirty minutes after sunset, we turned on a halogen lamp (500 W) at a distance of 5 m from the array to observe pollinators. Illumination at this distance slightly increased brightness in experimental arrays and we then could follow the movements of hawkmoths with the video camera. This illumination appeared to have no recognizable effect on hawkmoth behavior, although we were unable to exclude this possibility. On the digital Hi-Vision monitor, we identified pollinator types and recorded sequence and time of flower visits for each individual pollinator. However, we could not determine whether the same individual pollinators may have had more than one foraging bout. To evaluate the pollinator preference, we counted the number of visits per flower per day and determined the frequency of visits to *H. fulva* (Hf) and the frequency of visits to F1, designated as *p* and *q* respectively below. Considering a possibility that different individual pollinators had any preference to different type flowers (see [54], [55], we compared an observed frequency of plant-to-plant movements in a different class (Hf–Hf, Hf–F1, F1–Hf or F1–F1) with the expectation under the average preference of swallowtail butterflies and hawkmoths in experiment 1; the expected pairwise transitions of Hf–Hf, Hf–F1, F1–Hf and F1–F1 are p^2^, pq, pq and q^2^, respectively. If pollinator movements between plants were significantly different from these expectations towards more homotypic plant-to-plant movements, it would provide evidence of floral constancy, or the tendency of individuals to focus on one floral type.

### Data Analysis and Statistics

We analyzed the effects of flower type (*H. fulva* or F1 hybrid) or trait (flower color and scent) on the number of visitations per flower per day by using generalized linear mixed models (GLMMs) using poisson distribution and log link. Next, we added the height of a flower stem (from the ground to the top of an inflorescence) to an additional explanatory variable, because it was likely to affect pollinator visitation [56]. In experiment 1, flower type and the height of the flower stem were used as explanatory variables. In experiment 2, the color chart score, the intensity of scent, the height of the flower stem and the interaction between flower color and intensity of scent were used as explanatory variables. If the interaction was not significant, we excluded the interaction from these variables and used only the flower color, the intensity of scent, the height of flower stem and the observation date as explanatory variables. The observation date was included in GLMM analyses as a random effect. Then, in order to examine the consistency of trends, the statistical tests were performed for each observation day using generalized liner models (GLMs) with poisson family and log link. The number of visitations per flower per day was tested as dependant variable. In experiment 1, flower type and the height of the flower stem were used as explanatory variables. In experiment 2, the color chart score, the intensity of scent, the height of the flower stem and the interaction between flower color and intensity of scent were used as explanatory variables. In analyses for each observation day, we applied Bonfferroni corrections. In experiment 2, we analyzed the effect of the illumination on hawkmoth preference by performing a GLM analysis and likelihood ratio test. In this analysis, the number of visitations per flower per day was tested as dependant variable and the illumination (on/off), the color chart score, the intensity of scent and the height of the flower stem were used as explanatory variables.

We determined whether the pollinator’s plant-to-plant movements were predicted by their single-visit preferences using the *G*-test with Williams’ correction ( [57], p. 706). All statistical tests were performed with the computer software R (ver. 2.6.0).
